# Experiments with Iodine

**Published:** 1860-12

**Authors:** B. Woodward

**Affiliations:** Galesburg, Ill.


THE CHICAGO
MEDICAL JOURNAL.

VOL. III. December, 1860. NO. 12.

### Original Communications.

### ARTICLE 1.

### EXPERIMENTS WITH IODINE,

### AS AN ANIDOTE TO THE VENOM OF SERPENTS, AND CERTAIN OTHER ANIMAL POISONS.

BY B. WOODWARD, M. D., GALESBURG, ILL.

The paper Dr. S. W. Weil Mitchell, read before the Academy of Natural Sciences, at Philadelphia, October 16th, takes the ground that, "there appears to be no absolute antidote to the virus of the rattlesnake."

If by an "absolute antidote" the Doctor means, a substance which when brought in contact with the poison shall so neutralize it as to render it harmless, I must beg leave to differ from him. If he means "no substance which shall save the patient in every case," he certainly is correct. The ferri oxydum hydratum is an absolute antidote to arsenic if administered quickly and in sufficient quantities, forming as it does an insoluble arseniate of iron. Chalk is, in the same way, an antidote to oxalic acid, but the patients may be killed by these poisons; absorption having taken place to a great extent before the antidotes were administered. So with the rattlesnake. Iodine, as I shall endeavor to show, is a perfect antidote to it; still it may be that the patient may die from its effects, in consequence of the remedy not being used soon enough, or in sufficient quantities. In 1846, Dr. Whitmire first brought before the profession the value of Iodine as an antidote to the rattlesnake. In 1848 Dr. Harwood, through the *N. W. Med. and Surg. Journal* claims also to have used the remedy. But it was not until the publication of Prof. Daniel Brainard's experiments before the Academy of Sciences, that the true antidotal powers of iodine were established. These experiments were with curare, and the virus of the rattlesnake. It may be objected, that curare is a vegetable and not an animal poison. Though this is the view Dunglison takes of it, naturalists are divided on the question; some claiming it to be of animal origin, and others of vegetable. Be this as it may, those experiments fully demonstrated the power of iodine over carare.

In the winter of 1856 I had the pleasure of witnessing the same series of experiments, by Dr. Brainard.

In the July No. of the *Northwestern Medical and Surgical Journal*, I reported a case of successful treatment of rattlesnake bite by iodine. In that case the child, 12 years old. had been bitten several hours before I saw her, in the ankle. When I saw her the whole limb and side was very much swollen and discolored; tongue swelled; pulse 140; constant vomiting of green slime, abdomen tympanitic, she was delirious. She was first treated with morphia in ½ gr. doses, to allay the vomiting and quiet the brain. The limb and every part of the side was scarified and dotted with tr. iodine and as soon as the stomach would bear it, she took a teaspoonful every hour, of solut. of iod. potassa, 20 grs. to the ounce of water. The dotting with the iodine was repeated every six hours. Under this treatment she rapidly recovered.

In order to test the value of iodine in neutralizing animal poisons, I have, at different times, instituted experiments to that end.

The first series was in 1855, with the virus of the rattlesnake. Having procured a fine specimen of the massassauger, or black rattlesnake, I carefully dissected out the poison sacs at the roots of the fangs.

1st. With a needle dipped in the poison, I pricked the ear of a kitten about two months old. The animal immediately gave evidence of great pain, the head swelled, and in two hours she was dead.

2d. A drop of the poison was mixed, on a piece of glass, with a drop of tinc. iodine U. S. P. With this, another kitten was pricked twice through the ear. She gave no evidence of pain except scratching her ear for a minute or two. In half an hour she was again pricked in the ear with the clear poison. Almost instantly she cried out with pain. I then scraped the surface of the wounded ear, and poured on tr. iodine. The pain seemed last but a short time; the ear swelled slightly, but in two hours the cat was well, except that she had a sore ear.

3d. Another cat was wounded in the foot with a knife armed with the poison, and left for fifteen minutes. At the end of that time she was in agony. The iodine was poured on the foot and leg, but she died in an hour and a half.

4th. A kitten was wounded on the inside of the thigh, with a poisoned knife, and instantly tr. iodine was poured on. She gave no evidence of poisoning, though she was lame for two days.

5th. With a fine syringe I injected one drop of the virus which had beeu mixed with two drops of tr. iodine, under the skin of the leg of another cat. She was soon in great pain, and the limb became swelled. I scarified the limb and poured on tr. iodine; though she suffered for some hours she recovered.

*Virus of Spiders.*—I took a large yellow spider and made him bite my arm. There was instantly great pain. With a lancet I scarified around the wound and poured on tr. iodine. The pain soon abated. With a pair of forceps I drew out the poisonous fangs of the insect, and with one in its natural state, pricked my arm; there was great pain for a few minutes when I rubbed the part well with tr. iodine, and the pain abated. I now dipped the other fang in the tr. and pricked myself with it. There was neither pain nor swelling.

By repeated experiments I have found that the action of vaccine virus may be arrested twenty-four hours after its insertion, by the application of tinct. iodine; and that the virus will not act at all, if mixed with the iodine. Bosquet found that chloride of soda would not destroy the vaccine virus.

So far as stimulants and alkalis are of service, my own experience has not been in their favor, though it is true stimulants may be of use in sustaining the patient under the prostrating effects of the poison.

During a twelve years' residence on the prairies of Illinois, where these serpents are plentiful, it has frequently occurred to me to be called to treat their bites. In no case has it proved fatal where the iodine has been faithfully used locally and constitutionally.

I think I am correct in saying that in the published treatment of Dr. Wainwright, of New York, who died from the effects of the bite of a crotalus, iodine was not alluded to.

There is one other form of animal poison in which I have frequently (at least four times) found the iodine a perfect antidote. I refer to septic poison from a dead body. In one of these cases I was removing the abdominal viscera from the body a woman who had died of puerperal peritonitis, when I cut my hand with the scalpel. My friend and relative, Dr. B. Durham, of Chicago, immediately poured on some tr. of iodine, and I got no harm.

In July, 1858, while making a post mortem examination of the body of a man who had died twenty-four hours previously and which was in a rapid state of decomposition. My then partner, Dr. McCurdy, accidentally pricked my hand with a tenaculum which we were using. Though I sucked the well at the time, in about twelve hours my hand became very painful, the pain passing up my arm. Dr. McCurdy scarified the arm well in the track of the painful lymphatics, and then dotted with saturated tr. of iodine and took 10 grs. of iod. potassa every four hours till I had taken 70 grains, the arm was no pain or trouble. With regard to syphilitic virus I have found the saturated tr. of iodine to act promptly in arresting the disease, by bathing a *fresh* chancre with it, but where there was the least *constitutional* evidence of the disease, as bubo, no good results were obtained. I prefer the iodine treatment of recent chancre to nit. silver as the fluid penetrates all the tissues in the region of the sore.

Since the publication of Dr. Brainard's paper in the *Gazette des Hopitaux*, and its re-publication in this country, I have seen no record of any experiments going to throw doubt on the question. From which I have tested of the efficacy of the iodine treatment, I believe others will be equally well satisfied if they will give it a fair trial.

